# A Systematic Review on Postoperative Antibiotic Prophylaxis after Pediatric and Adult Male Urethral Reconstruction

**DOI:** 10.3390/jcm12196162

**Published:** 2023-09-24

**Authors:** Łukasz Białek, Marta Rydzińska, Malte W. Vetterlein, Jakub Dobruch, Michał A. Skrzypczyk

**Affiliations:** 1 Department of Urology, Centre for Postgraduate Medical Education, 01-813 Warsaw, Poland; 2 Department of Urology, University Medical Center Hamburg-Eppendorf, 20251 Hamburg, Germany

**Keywords:** urethral reconstruction, urethral stricture, hypospadias, antibiotic prophylaxis, urinary tract infection, wound infection

## Abstract

In the era of antibiotic overuse and increasing antibiotic resistance, there is a gap in evidence regarding antibiotic stewardship, and in particular, perioperative antibiotic prophylaxis after urethral reconstruction. The aim of this systematic review was to evaluate the effectiveness and relevance of postoperative antibiotic prophylaxis after male pediatric and adult urethral reconstruction. An online search of MEDLINE database via PubMed was performed. The systematic review was registered in PROSPERO (CRD42022348555) and was conducted according to the PRISMA guidelines and AMSTAR 2 checklist. A narrative synthesis of included studies was performed. After the screening of 1176 publications, six studies regarding antibiotic prophylaxis after hypospadias reconstruction and two studies regarding antibiotic prophylaxis after urethroplasty in adults were eligible to be included in the systematic review. All but one of the studies on hypospadias repair showed no benefit from postoperative antibiotic prophylaxis. The level of evidence on postoperative antibiotic prophylaxis after urethroplasty in adults is low. Neither of the two studies included in the review showed a benefit from antibiotic use. Postoperative prophylaxis after hypospadias repair is not effective in preventing urinary tract infections and wound infections. It seems that the use of postoperative prophylaxis after urethroplasty in adults is also not beneficial, but there is a high need for high-quality scientific data.

## 1. Background

Urethral stricture disease (US) is a common urological condition in men. Although rigorous epidemiologic data is sparse, the existing papers report an incidence varying between 0.6 and 1.4 percent. Urethral strictures can occur throughout the entire length of the urethra but mainly involve the anterior urethra and, in particular, the bulbar segment. Reconstructive urethral surgery is the gold standard in the treatment of US in males as well as other urethral abnormalities such as urethrocutaneous fistulae, diverticula, and congenital defects, including hypospadias [1,2]. Endoluminal treatment of anterior US by urethral dilatation or direct visual internal urethrotomy (DVIU) brings rapid improvement in micturition quality but is associated with a very high rate of stricture recurrence in up to 92% of patients [3]. Reconstructive surgery is the only option to treat hypospadias and restore physiological lower urinary tract anatomy and functionality as well as cosmetic appearance [4].

There is proof that up to 50% of prescribed antibiotics are used unnecessarily or inadequately [5]. Unnecessary and improper use of antimicrobials contributes to the development of antibiotic resistance, which poses a threat to public health [6]. Additionally, this raises the likelihood of adverse medication effects.

Urinary tract infection (UTI) and wound infection are common after urethroplasty and may lead to surgical failure or distant complications [7]. Thus, both the European Association of Urology (EAU) and American Urological Association (AUA) guidelines recommend intraoperative antibiotic prophylaxis [2,7]. Moreover, according to recent surveys, the majority of leading reconstructive urologists involved in urethroplasty recommend prolonged oral antibiotic prophylaxis lasting until the catheter is removed [8,9]. However, there is no clear answer as to whether prolonged antibiotic use after urethral reconstruction reduces the rate of UTIs and wound infection. Moreover, according to the study by Hanasaki et al., it seems that proactive antibiotic discontinuation seems to have no negative impact on postoperative morbidity [10]. Additionally, the panel of AUA guideline authors recognizes that the question of the optimal duration of antibiotic therapy after urethroplasty remains one of the important issues for future consideration and research [2]. For this reason, we decided to perform a systematic review of the literature to evaluate the effectiveness and relevance of postoperative antibiotic prophylaxis after urethral reconstruction.

## 2. Material and Methods

The systematic review was performed in accordance with the Preferred Reporting Items for Systematic Reviews and Meta-Analyses (PRISMA) statement and the Cochrane Handbook for Systematic Reviews of Interventions. The study protocol was registered with PROSPERO (CRD42022348555).

A systematic search was conducted independently by two authors (Ł.B. and M.R.) through the PubMed electronic database according to the PRISMA statement [11]. The last search was performed on 1 September 2022. The search query was (*antibiotics OR anti-bacterial agents OR antibiotic prophylaxis OR urinary tract infection OR wound infection*) *AND* (*urethroplasty OR urethral reconstruction OR urethral stricture*). The search included articles without time limitations. Only publications in English were considered and evidence was limited to human data. Moreover, all references within retrieved articles were screened for additional relevant articles.

The inclusion criteria for this systematic review were as follows: randomized controlled trial, prospective or retrospective cohort study, male urethral reconstruction, and full-text publication in English. Reviews, case reports, letters to the editors, conference abstracts with no full text, and commentaries were excluded.

After the removal of duplicates, two authors (Ł.B. and M.R.) independently evaluated the titles and abstracts of the retrieved records. All potentially eligible studies were evaluated as full text if available. Disagreements were resolved by consultation with the senior author (M.S.).

## 3. Evidence Synthesis

**Figure 1:**
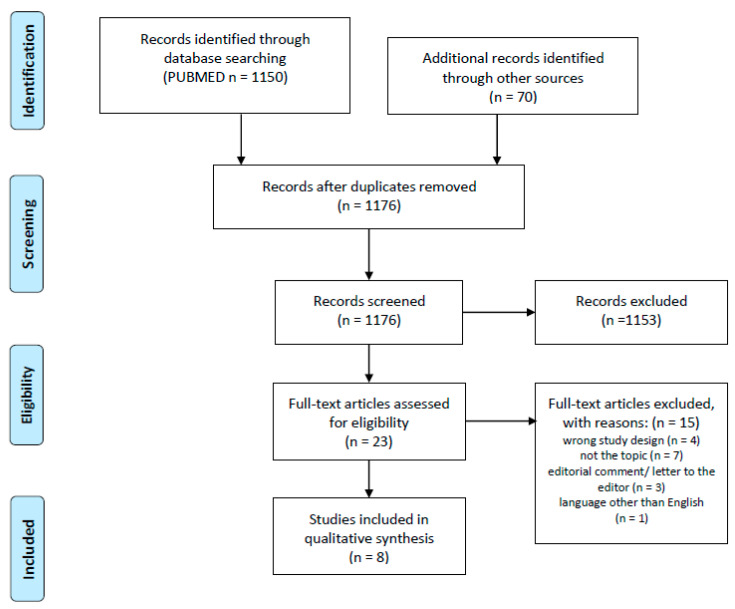
PRISMA flow diagram.

After the screening of 1176 publications, eight were eligible to be included in this systematic review. Figure 1 shows the selection process of the study in accordance with PRISMA. Out of all the publications, there were twenty-three assessed in the full-text, fifteen of which were eventually excluded due to wrong study design (four studies), not meeting the topic of the systematic review (seven studies), editorial comments or letter to the editor (three studies), and language other than English (one study). Out of eight studies included into the systematic review, five were from the US, one was from Israel, one from Canada, and one from Sweden. There was one double-blind, placebo-controlled multi-institutional randomized trial [12], two randomized, open-label single-center trials [13,14], and three prospective single-center studies [15,16,17], which dealt with postoperative antibiotic prophylaxis after urethral reconstructions due to hypospadias in children. The two remaining studies were retrospective cohort studies regarding antibiotic prophylaxis after urethroplasty in adults [18,19].

### 3.1. Postoperative Antibiotic Prophylaxis after Hypospadias Repair

Six studies assessed postoperative antibiotic prophylaxis after hypospadias repair (Table 1). The studies varied significantly in their methodology. In some of them, all patients received perioperative antibiotic prophylaxis, which, however, consisted of different agents [15,16,17]. In one study, the control group received an antibiotic agent around the time of catheter removal [16], while in other studies, the control group received no postoperative antibiotic prophylaxis [12,13,14,15,16]. The antibiotic prophylaxis regimens themselves also differed in the cited studies—antibiotics were given for 10 days, or until the catheter/stent was removed, or even longer, several days after catheter/stent removal. The most commonly used drug was trimethoprim-sulfamethoxazole (TMP/SMX). In all studies, UTI was diagnosed on the basis of positive urine culture and lower urinary tract symptoms or fever. None of the studies showed a significantly higher rate of wound infections in patients who did not receive postoperative prophylaxis. All but one of the studies also failed to prove a higher rate of UTIs, urethrocutaneous fistula formation, or external outlet stenosis [12,13,14,16,17]. Only in the study by Meir et al. were the rates of UTIs, urethrocutaneous fistula formation, and meatal stenosis significantly higher in patients who did not receive postoperative antibiotic prophylaxis [15].

### 3.2. Postoperative Antibiotic Prophylaxis after Urethroplasty

Postoperative antibiotic prophylaxis after urethroplasty was a subject of two studies (Table 2). Both studies were retrospective analyses of the effect of antibiotic prophylaxis on the rate of UTIs and wound infections, due to a change in internal policies of the institutions that participated in the studies. In the study by Baas et al. [18], the first group of patients received an antibiotic (not specified) for three weeks until catheter removal. The second group in postoperative prophylaxis received an antibiotic only for three days around the day of catheter removal. There was no statistically significant difference in the percentage of patients who developed UTI or wound infection, and overall, the percentage of these events was low (UTI: 6.7% in group 1, and 12% in group 2; wound infection: 3.3% and 1.7%, respectively). The paper published by Kim et al. was a summary of a multicenter study that included patients treated with urethroplasty at eleven American centers over a two-year period [19]. Patients who were treated in the first year (group A) received an antibiotic agent (mainly nitrofurantoin) until catheter removal and additionally ciprofloxacin or TMP/SMX around catheter removal. Patients treated in the second year (group B) received ciprofloxacin or TMP/SMX only around catheter removal as postoperative prophylaxis. Again, there were no statistically significant differences in the percentage of patients who developed UTI or wound infection, and the percentage of these patients was generally similar to the previous study (UTI: 6.7% in group A and 3.9% in group B; wound infection: 4.1% and 3.7%, respectively). The authors also performed a multivariate analysis that attempted to find potential predictors of UTIs and wound infections—despite the inclusion of several parameters from the univariate analysis, the multivariate analysis failed to identify such predictors.

## 4. Discussion

In the face of growing antibiotic resistance, every possible effort should be made to promote adequate and reasonable use of antimicrobial therapy. Single-dose periprocedural antibiotic prophylaxis is currently recommended for all patients undergoing open urological surgery [20]. However, the risk of bacteriuria increases by 3–8% for each consecutive day of catheterization, and the duration of catheterization has been proven to be the most important risk factor for catheter-associated UTIs [21]. Thus, the most effective strategies for reducing such infections are limiting catheterization or promptly removing catheters when they are no longer indicated. In some clinical situations, however, leaving the transurethral catheter is indispensable. One of those is urethral reconstruction.

A Cochrane review of antibiotic prophylaxis for short-term urinary catheter bladder drainage points out that there is a limited data suggesting a reduced rate of bacteriuria and symptomatic UTI in surgical patients receiving antibiotic prophylaxis who undergo bladder drainage for at least 24 h postoperatively [22]. Also, more recent meta-analyses suggest that patients with catheters being removed might benefit from antibiotic prophylaxis as a result of fewer subsequent UTIs [23,24]. It is, however, important to point out that in the context of urethral reconstruction, the urinary catheter not only ensures the urine outflow from the bladder, but also passes directly through the surgical site, making the latter potentially more vulnerable to infection. A similar situation occurs in patients undergoing radical prostatectomy, where the catheter splints the vesico-urethral anastomosis. However, the data available in the literature on antibiotic prophylaxis in these patients are not consistent in terms of reducing UTIs [25,26]. On the other hand, for many other major surgeries, limiting the duration of perioperative antibiotic prophylaxis is not associated with an increased risk of infectious complications and has a beneficial effect in terms of antimicrobial-associated adverse events [27,28,29]. In the light of the lack of specific recommendations, most urologists performing urethral reconstruction recommend to their patients prolonged antibiotic prophylaxis both during their hospital stay and their subsequent recovery at home [8]. This may be due to the fact that most of the complications experienced by patients undergoing urethral reconstruction are infectious, hence surgeons giving prolonged antibiotic prophylaxis aim at reducing them [30]. There are also isolated reports on giving only targeted antibiotic therapy, with urine culture testing pre-, peri-, or postoperatively trying to reduce standard postoperative prophylaxis [31]. On the other hand, complications such as wound infection or UTI are rarely reported in the pediatric population undergoing hypospadias repair [32]. Given the lack of distinct guidelines and the importance of the problem, we decided to perform a systematic review on the impact of postoperative antibiotic prophylaxis.

We were able to bring to light fairly strong evidence regarding the use of postoperative antibiotic prophylaxis after hypospadias repair in children. According to Hsieh et al., pediatric urologists in the US employ antibiotic prophylaxis in clinical practice at significantly different rates [33]. In hypospadias repair with a postoperative catheter only, 77% used perioperative antibiotics and 91% used postoperative antibiotics. As summarized in this systematic review, several studies (including prospective and randomized trials) have been published on this topic, and in all but one (which was neither a double-blinded nor placebo-controlled study), there was no significant difference in the percentage of patients who experienced UTI, urethrocutaneous fistulae, meatal stenosis, or wound infection. Previously, Chua et al. published a systematic review and meta-analysis on antibiotics in hypospadias repair [34]. While the authors collectively evaluated both preoperative and postoperative antibiotic prophylaxis studies, we focused exclusively and entirely on postoperative antibiotic prophylaxis in our review. In addition, since the publication by Chua et al., the final publication on the double-blind, placebo-controlled, multi-institutional randomized trial PROPHY has been published, which deserves attention, given the type of study that is still uncommon in reconstructive urology [12]. It is noteworthy, however, that a major limitation of this study is the smaller-than-desired sample size secondary to inadequate recruitment, making the study possibly underpowered to reliably detect a difference in outcomes. Moreover, in all the studies presented, the majority were patients with primary hypospadias and distal or mid-shaft hypospadias. Hence, caution should be taken when extrapolating these results to redo cases and proximal hypospadias. Nevertheless, as a conclusion, we can, therefore, assume that the administration of postoperative antibiotic prophylaxis does not result in additional reduction of complications and should, therefore, not be routinely used.

Within the topic of postoperative antibiotic prophylaxis after urethroplasty, we only found two studies evaluating infectious complications. Neither of them was a randomized trial, so the level of evidence is low. Antibiotic prophylaxis was not reported to reduce the rate of UTI or wound infection in any of the studies. However, even patients in the “no postoperative antibiotic prophylaxis” groups received antibiotics around the time of catheter removal. The duration of catheterization after urethroplasty is a matter of debate [35,36], yet in the studies included in the systematic review, the effect of transurethral catheter and/or suprapubic catheter maintenance time on the rate of infectious complications was also not analyzed. In the study by Baas et al., all patients had the catheter removed three weeks after surgery. However, there is no information on the maintenance of the possible cystostomy [18]. In the multicenter study by Kim et al., the catheter was removed per usual clinical practice, and the authors themselves note that one of the limitations of their study is the non-standardized presence of the suprapubic catheter and the duration of catheterization [19]. The unknown duration of cystostomy maintenance and the varying duration of transurethral catheter maintenance are among some of the most significant limitations of the mentioned studies. One should also notice that the authors of the aforementioned studies do not address the possibility of performing urine culture preoperatively and its possible impact on prolonging antibiotic prophylaxis after the surgery, as we believe may be of great importance.

It is also worth mentioning that we currently do not have reliable data on the possible effect of postoperative antibiotic prophylaxis after urethroplasty on the recurrence of US or patient-reported treatment satisfaction after surgery. None of the studies enclosed in the systematic review assessed the possible effect of antibiotic prophylaxis on stricture recurrence. On the other hand, preoperative bacteriuria may increase the risk of recurrence, according to the study by Roehrborn et al. [37]. It is notable, however, that a proposal has recently been published to create distinct stricture-fecta criteria that could account scientifically for the success of urethroplasty [38]. Lack of postoperative complications, including infectious complications, is one of the initially proposed items.

In conclusion, the administration of postoperative antibiotic prophylaxis after pediatric hypospadias repair does not result in additional reduction of complications and should, therefore, not be routinely used. The postoperative antibiotic prophylaxis after urethral reconstruction in adults does not seem to be beneficial in terms of preventing UTIs and wound infections; however, the quality of evidence in this topic is low. Therefore, there is a great need for high-quality scientific data such as a randomized trial that could unequivocally assess the appropriateness of postoperative antibiotic prophylaxis in the prevention of UTIs, postoperative wound infections, and most importantly, their impact on urethral stricture recurrence and treatment satisfaction.

## Figures and Tables

**Table 1:** Summary of studies assessing antibiotic prophylaxis after hypospadias repair in children.

Study	Study Type	Patients’ Age	Surgery Type	Perioperative Antibiotic Prophylaxis	Postoperative Antibiotic Prophylaxis Pattern	Number of Patients	UTI Criteria	UTI	Wound Infection	Urethrocutaneous Fistulae	Meatal Stenosis
Meir, 2004 [15]	Prospective, single center	2.3	TIP hypospadias repair	Cefonicid iv., 1 dose	Cephalexin 3× daily Up to 2 days after catheter removal	52	Positive culture AND fever	3/52 (5.8%)	n/a	3/52 (5.8%)	0/52 (0%)
					No prophylaxis	49		12/49 (24.5%)	n/a	9/49 (18.4%)	4/49 (8.2%)
Kanaroglou, 2013 [16]	Prospective, single center	1.1	Hypospadias repair: TIP or glanular approximation or staged preputial flap/graft	Cefazolin iv.	TMP 2 mg/kg daily until catheter removal	78	Positive culture AND symptoms	0/78 (0%)	0/78 (0%)	8/77 (10.3%)	4/77 (5.2%)
		1.4			No prophylaxis	71		0/71 (0%)	0/71 (0%)	4/59 (6.8%)	1/58 (1.7%)
Zeiai, 2016 [17]	Prospective, single center	2	Primary hypospadias repair: TIP with postoperative indwelling stent for 7 days	TMP-SMX	TMP-SMX 2× daily until 3–7 days after stent removal	58	Positive culture AND fever	1/58 (1.7%)	2/58 (3.4%)	5/58 (8.6%)	1/58 (1.7%)
		1.5			TMP-SMX one dose at the removal of the stent	55		1/55 (1.8%)	1/55 (1.8%)	2/55 (3.6%)	0/55 (0%)
Canon, 2018 [14]	Randomized, open-label, single center	0.7	Primary distal hypospadias repair with open urethral stent drainage	none	TMP-SMX OR nitrofurantoin OR cephalexin	24	Positive culture AND symptoms	1/24 (4.2%)	0/24 (0%)	1/24 (4.2%)	0/24 (0%)
		0.9			No prophylaxis	24		0/24 (0%)	0/24 (0%)	1/24 (4.2%)	1/24 (4.2%)
Roth, 2018 [13]	Randomized, open-label, single center	0.8	Primary mid-to-distal shaft hypospadias repair	none	TMP-SMX until catheter removal	35	Positive culture AND symptoms	0/35 (0%)	2/35 (5.7%)	1/35 (2.9%)	1/35 (2.9%)
		0.9			No prophylaxis	31		0/31 (0%)	1/31 (3.2%)	2/31 (6.5%)	2/31 (6.5%)
Faasse, 2022 [12]	Double-blind, placebo-controlled, multi-institutional randomized trial	0.8	Midshaft-to-distal single-stage hypospadias repair	At surgeon’s discretion	TMP-SMX 2× daily for 10 days	45	Positive culture AND symptoms	2/45 (4.4%)	1/45 (2.2%)	5/45 (11.1%)	1/45 (2.2%)
					Placebo	48		3/48 (6.2%)	0/48 (0%)	1/48 (2.1%)	1/48 (2.1%)

TMP—trimethoprim; TMP-SMX—trimethoprim-sulfamethoxazole; UTI—urinary tract infection; TIP—tabularized incised plate.

**Table 2:** Summary of studies assessing antibiotic prophylaxis after urethral reconstruction in adults.

Study	Study Type	Patients’ Age	Surgery Type	Perioperative Antibiotic Prophylaxis	Postoperative Antibiotic Prophylaxis Pattern	Number of Patients	UTI Criteria	UTI	Wound Infection
Baas, 2021 [18]	Retrospective, single center	52	Urethroplasties	Cefazolin 2 g OR Ciprofloxacin 500 mg	Antibiotic prophylaxis for 3 weeks until the catheter removal	60	Positive culture OR LUTS treated empirically	4/60 (6.7%)	2/60 (3.3%)
		53			Antibiotics for 3 days around the catheter removal	60		7/60 (11.7%)	1/60 (1.7%)
Kim, 2022 [19]	Retrospective, multi-institutional	50	Urethroplasties	Cephalosporin iv. for 24–48 h OR culture-specific 3–5 days prior to surgery	Nitrofurantoin 100 mg 2× daily (or cefalexin) until the catheter removal PLUS Ciprofloxacin or TMP-SMX (2 doses) around the catheter removal	390	100 K CFU/mL AND symptoms	26/390 (6.7%)	16/390 (4.1%)
					Ciprofloxacin or TMP-SMX (2 doses) around the catheter removal	510		20/510 (3.9%)	19/510 (3.7%)

TMP-SMX—trimethoprim-sulfamethoxazole; UTI—urinary tract infection.
